# Notes and News

**Published:** 1901-11-02

**Authors:** 


### Notes and News.

The number of small-pox cases in London have been increasing. The largest number have recently come from Bermondsey.

The committee of the North London Hospital for Consumption, Mount Vernon, Hampstead, has received a donation of £1,000 to name a bed "In memory of the late Mrs. Henry Claudet, who died at Cannes."

The newest inoculation treatment proposed is that of Professor Löffler, of Greifswald, who recommends that cancer patients should be inoculated with the poison of the malaria mosquito. He supports his theory by the fact that cancer is absent from malarial districts.

There are no patients requiring more care than the chronic and incurable. This is above all the case with children who, with proper care and nourishment, frequently are restored to the ranks of the healthy. The call for accommodation is very great and the supply very small. Liverpool is doing its best to provide for its population by establishing a new hospital near West Kirby, and appeals are being made to obtain the required funds. One generous sympathiser has offered £5,000.

In delivering the second annual Huxley Memorial Lecture of the Anthropological Institute on Tuesday, Mr. Francis Galton discussed the question of the "Possible improvement of the human breed under existing conditions of law and sentiment." The aim of the lecture was to give a scientific basis to the problem of race improvement, and Mr. Galton showed that if one could arrive at a numerical or monetary value of different lives much could be done to encourage parentage among those who were shown to be of greatest value to their community. "It might," he said, "become an avowed object of noble families to gather fine specimens of humanity round them, just as it was to produce fine breeds of cattle and so forth, which were costly in money but repaid in satisfaction."

The Post Office collection on behalf of the Prince of Wales's Fund calls for admiration. No less than £152 is collected in annual subscriptions, and the donations last year amounted to upwards of £25 in addition.

The authorities of Addenbrooke's Hospital, Cambridge, report the finances of the institution to be in a worse condition than ever. The deficit on income is more than double what it was. The Governors were told at the recent meeting that either £25,000 must be secured as an endowment, or the number of beds must be reduced. It was not possible, they were told, to secure a sufficient income from the county of Cambridge.

Attention is being called to the sanitary condition of Leicester Infirmary. Professor Corfield has been called in to report, and the medical staff are very decided in their opinion that his recommendations should be put into effect. The Board, however, have expressed the view that much that has been recommended is unnecessary, and that the infirmary is by no means in a bad condition; in fact, as the chairman put it, the sanitary condition of the infirmary was far better than that of any private house in Leicester.

We have become so familiar with the presence of the plague in India that little attention is paid to statistics, which at one time caused serious uneasiness in this country. Yet the devastation throughout India at the present time is really very great, and there is no evidence of that steady diminution which might be looked for in response to the sanitory measures that have been put into execution, and which continue to be the subject of careful study throughout the Empire. The last return gave an appalling death list of 6,386 during one week. Last year the record was 593 only for the same period. Cholera and leprosy add to the very high Indian death rate, but they fall into the background in the face of the most dreaded epidemic.

The official account of the case of the late President McKinley which has now been issued, although it adds but little of interest to the information which had already been made public, is a very complete and elaborate document, and one which ought, we think, to satisfy the American public that their President was treated with the highest skill, and that, whatever may have been the exact pathological sequence of events or the precise cause of his death, nothing was omitted which would have been in the least degree likely to have averted the fatal issue. The report commences by stating the fact of and the hour of the assassination, and from that time forward describes with the greatest care and minuteness all the steps taken until death occurred. It is followed by a report of the autopsy and by one of the bacteriological examination. These several reports have all been drawn up with great care, and the whole document is not only a most interesting record of a peculiarly interesting case, looking at it for the moment from the purely professional point of view, but is also a record which will live in history, as showing the high position attained by American surgery.

